# Classification of angioedema types using decision tree modeling

**DOI:** 10.3389/fimmu.2025.1697143

**Published:** 2026-01-12

**Authors:** Felix Aulenbacher, Annika Gutsche, Henriette Farkas, Kinga Viktória Kőhalmi, Emek Kocatürk, Emel Aygören-Pürsün, Ludovic Martin, Hilary Longhurst, Petra Staubach, Andrea Zanichelli, Werner Aberer, Anette Bygum, Mignon van den Elzen, Thomas Buttgereit, Markus Magerl

**Affiliations:** 1 Angioedema Center of Reference and Excellence (ACARE), Institute of Allergology, Charité – Universitätsmedizin Berlin, corporate member of Freie Universität Berlin and Humboldt-Universität zu Berlin, Berlin, Germany; 2 Fraunhofer Institute for Translational Medicine and Pharmacology (ITMP), Immunology and Allergology, Berlin, Germany; 3 Hungarian Angioedema Center of Reference and Excellence, Department of Internal Medicine and Haematology, Semmelweis University, Budapest, Hungary; 4 Department of Rheumatology and Immunology, Semmelweis University, Budapest, Hungary; 5 Department of Dermatology, Bahçeşehir University School of Medicine, Istanbul, Türkiye; 6 Angioedema Center, Internal Medicine and Hemostaseology, Department for Children and Adolescents, University Hospital Frankfurt, Goethe University, Frankfurt, Germany; 7 Department of Dermatology, Angers University Hospital, Angers, France; 8 Department of Immunology, Auckland City Hospital, Auckland, New Zealand; 9 Department of Medicine, University of Auckland, Auckland, New Zealand; 10 Department of Dermatology, University Medical Center Mainz, Mainz, Germany; 11 Department of Biomedical Sciences for Health, University of Milan, Milan, Italy; 12 Operative Unit of Medicine, Angioedema Center, IRCCS Policlinico San Donato, San Donato Milanese, Milan, Italy; 13 Department of Dermatology, Medical University of Graz, Graz, Austria; 14 Clinical Institute, University of Southern Denmark, Odense, Denmark; 15 Department of Dermatology/Allergology, University Medical Center Utrecht, Utrecht University, Utrecht, Netherlands

**Keywords:** angioedema, hereditary angioedema, bradykinin-mediated angioedema, mast cell-mediated angioedema, machine learning, Random Forest, diagnostic classification, clinical decision support

## Abstract

**Introduction:**

All angioedema (AE) presents with transient, localized swelling; however, the underlying causes, prognosis, and treatments vary significantly. Consequently, identifying a specific AE type is challenging.

**Methods:**

We aimed to apply a machine learning (ML) model to improve AE diagnosis. Random forest (RF) ML was used to create a prediction model for diagnosing correct AE types. Development comprised a literature search to establish AE's clinical characteristics, developing and translating questions in collaboration with 12 European AE centers, and selecting, testing, validating and optimizing the established ML model. Analysis included 342 specialist-diagnosed patients with one of six AE types.

**Results:**

The final optimized RF model correctly identified AE types with true positive rates of up to 94% in hereditary AE due to C1 inhibitor deficiency (C1INH), with a Percentage Accuracy of 89·2% and a Kappa value of 81·8% across the six AE types, with a high agreement with the diagnoses made by experts.

**Discussion:**

This is the first ever reported ML algorithm designed to pre-assess to aid AE diagnosis.

## 1 Introduction

Angioedema (AE) manifests with localized, intermittent, self-limiting subcutaneous and/or submucosal tissue swelling (1). It involves the dermis and vascular subcutis or mucosal membranes and can last between a few hours and a few days. AE most often occurs spontaneously, but can be induced by specific triggers (2). Individuals who experience AE often do so repeatedly rather than having an isolated episode, and so experience recurrent AE. The diagnosis of recurrent AE is based on the clinical presentation (including photo documentation of swellings) and the patient’s medical history. Several forms of AE exist, depending on the underlying pathogenesis. They can be divided into two frequent categories: mast cell-mediated AE (AE-MC) and bradykinin-mediated AE (AE-BK) (3). Other categories include AE due to vascular endothelium dysfunction (AE-VE), mast cell- or bradykinin-independent drug-induced AEs (AE-DI), and AE of unknown cause (AE-UNK) (3).

The most common form of AE, AE-MC, is due to chronic urticaria (AE-URT), defined as the recurrence of wheals, itch and/or angioedema for six weeks or longer (4). AE is present in around 45% of patients with chronic spontaneous urticaria (CSU) (5) and is associated with a high disease burden and long duration. In CSU, AE and wheals are induced by the degranulation of mast cells in the skin, which are co-localized with sensory nerves and blood vessels; their activation and release of pro-inflammatory mediators, particularly histamine, induce oedema and erythema.

AE-BK occurs in hereditary AE (HAE) with or without C1INH deficiency (HAE-C1INH, e.g. HAE-PLG, HAE-FXII), acquired AE due to C1INH deficiency (AAE-C1INH), or AE-DI due to intake of drugs targeting the renin-angiotensin-aldosterone system (RAAS, i.e. AE-ACEI, AE-Sartan and AE-Gliptin, presumably BK-driven) (1). Most of these types of AE are usually present without wheals and are considered rare diseases, except AE-ACEI, due to the high usage of ACEI. BK promotes vasodilation via the BK B2 receptor pathway. The kinin cascade generates BK, and C1INH regulates the cascade. Dysregulation of BK generation or impaired metabolism of BK can lead to excess BK, which may result in AE.

Since treatments and prognoses vary across AE types, accurately identifying the specific type in each patient is essential. However, this task is particularly challenging because the swellings associated with different AE types appear similar, and most lack definitive biomarkers. As a result, the delay in diagnosing the correct AE type is protracted; patients with HAE with normal C1INH values (HAE-nC1INH) typically experience delays of over ten years until diagnosis (6).

The rate of misdiagnosis is high, with incorrect diagnoses often including allergies or appendicitis (7–9). Consequently, inappropriate treatments such as antihistamines, epinephrine, or immunosuppressive therapies are administered, or even surgical procedures (6, 10). These missteps lack clinical utility and can significantly harm the patient (11, 12).

We used an existing ML function from an R package to build a prediction model that classifies AE types. We aimed to test the applicability of the model in predicting the correct AE types to assist physicians during the diagnosis prospectively. To do this, we collected clinical information from patients with various types of AE, generated a questionnaire to distinguish between the types of AE and developed an Artificial Intelligence algorithm. The algorithm learns to make predictions by identifying patterns in patient responses, based on previously collected and labeled data.

## 2 Materials and methods

The selection of an ML model for the diagnosis of AE involved three phases: 1) a literature search to establish the clinical characteristics of different types of AE, 2) the development and translation of questions in collaboration with 12 European AE centers, and 3) selection, testing, validation, and optimization of the established ML model.

### 2.1 AE nomenclature

At the time of drafting the study presented here, the classification and nomenclature of AE at that time were used. Since August 2024, a new consensus on the definition, acronyms, nomenclature, and classification of angioedema (DANCE) has been in effect (1). We have made every effort to align our manuscript with the current terminology (**Supplementary Table 1**).

### 2.2 Literature search

We conducted a non-systematic literature search to identify clinical features and characteristics that are typical or atypical for six different AE types, aiming to differentiate one from another: AE-MC (AE-URT), AE-BK (HAE-C1INH, HAE-nC1INH [i.e. HAE-FXII, HAE-PLG], and AAE-C1INH), AE-ACEI, and idiopathic (non-histaminergic) AE (AE-UNK). The items found were extracted from the literature to prove there is evidence for the assumed differences. Search terms and search strategies were adapted according to the AE type; the search results were used to meta-analyze the existing literature for potential clinical markers with high differentiation grades (see next section; **Table 1**).

**Table 1:** The final 10 questions used in the questionnaire.

Question number	Topic	Question
1	Medication history	Do you (or did you so in the last 6 months) take an ACE inhibitor? (ACE-inhibitors are drugs to treat heart or kidney diseases (e.g. high blood pressure) and their active substances are named with the ending -pril, e.g. captopril, ramipril, or enalapril).
2	Medication history	Do you take (or did you so in the last 6 months) a sartan or a gliptin? (Sartans are drugs to treat heart diseases (e.g. high blood pressure) and their active substances are named with the ending -sartan, e.g. candesartan, losartan, or valsartan Gliptins are drugs to treat diabetes and their active substances are named with the ending -gliptin, e.g. sitagliptin, saxagliptin, or linagliptin).
3	Symptom history	At what age (approximately) did the angioedema symptoms start? (Angioedema are usually skin colored, mostly hard to delimit, sometimes painful swelling of the skin or mucous membranes, for example of the eyelids, lips, tongue, hands or feet. In some patients angioedema can occur in the abdominal organs, leading to colicky pain, sometimes including vomiting or diarrhea. In other patients angioedema can occur in the throat, leading to breathing difficulties. All these forms of angioedema can last for several hours to days)
4	Family history	Do or did any other family members (related by blood) suffer also from recurrent angioedema?
5	Prodromal symptoms	Do or did you experience repeatedly symptoms other than angioedema (e.g. fatigue malaise, non-itchy skin rashes) approximately one hour or longer before the actual angioedema symptoms start?
6	Response to therapy	Do or did you experience repeatedly severe and extremely painful abdominal attacks?
7	Swellings	Did of the swellings in the past affect the tongue?
8	Swellings	Did or do the swellings last for 3 days or longer, until they resolve completely?
9	Occurrence of wheals	Do or did you suffer from recurrent itchy wheals? (Wheals are elevated, red and itchy skin eruptions that look like stinging nettle burns, see back of this sheet for an example)
10	Response to therapy	Are your symptoms of angioedema well controlled by the use of antihistamines or cortisone or omalizumab? (Antihistamines are antiallergic tablets, e.g. loratadine or cetirizine. Omalizumab (Xolair) is an every-4-weeks injection for the treatment of chronic urticaria)

The final 10 questions were included in the questionnaire.

### 2.3 Development of AE questions

The rationale for selecting the questions was based on the observation that different forms of recurrent angioedema have different clinical characteristics that are very typical for one or a few forms and completely atypical for others. For example, the onset of the first symptoms in HAE-C1INH is very typical in childhood and adolescence, in urticaria often in young and middle adulthood, and in AAE-C1INH in older age. A positive family history is expected in the vast majority of patients with HAE-C1INH, in the majority of patients with HAE-nC1INH, but almost never in patients with AE-URT or AE-ACEi. Recurrent wheals are reported by a majority of patients with AE-URT but rarely in other types of AE. The questions were selected according to this scheme based on their discriminatory power and formulated in such a way that patients can answer them easily, clearly, and directly from memory. An initial set of 11 draft questions was emailed to the heads of all participating centers, who were tasked with selecting and refining items for the questionnaire. The experts collaboratively reviewed the proposed questions, focusing on their wording, selection criteria, and overall relevance. This review process was conducted in parallel with a literature search, which helped identify distinguishing clinical features across AE subtypes. Final decisions on question inclusion were based on expert consensus regarding each item’s diagnostic usefulness during routine patient history-taking. Following expert input and analysis, 11 questions were developed in collaboration with 12 AE centers (**Table 1**). For example, do you suffer, or have you suffered from recurring wheals that itch? At what age (approximately) did you first experience angioedema? The original 11 questions used during data collection were reduced to 10, as questions 2 and 3 were merged into a single question before the ML process. It became apparent that the frequency of use was lower than initially assumed. Data analysis showed that gliptins play a minor role in triggering angioedema, and we found this only due to their low response frequency. The original questions 2) and 3) were: 2) Do you take (or did you in the last 6 months) a gliptin? 3) Do you take (or did you in the last 6 months) a sartan? The new combined question was: Are you taking (or have you been taking for the past 6 months) a sartan or a gliptin?

The questions were compiled into a paper-based questionnaire and distributed to patients receiving treatment at AE clinics in Angers, Berlin, Budapest, Frankfurt, Graz, Istanbul, Jönköping, London, Mainz, Milano, Odense, and Utrecht. Once a physician diagnosed the patient’s AE type in an AE center, patients answered questions anonymously without identifying any personal data.

### 2.4 Selection, testing, validation and optimization of the established ML model

#### 2.4.1 Data set

In total, 350 patients with one of the six different AE types were surveyed; an expert diagnosis was available for each. Only fully completed questionnaires were used to test the ML models. The information obtained from individual patient surveys matched the diagnosis established by their treating physician.

#### 2.4.2 Generation of the ML model based on default settings

The survey data was used to generate different standard, established ML models, which included the Linear Discriminant Analysis (LDA), Nonlinear Algorithms Classification and Regression Trees (CART [rpart]), k-nearest Neighbors (k-NN), Support Vector Machines with Radial Basis Function Kernel (SVMRadial), and the Random Forest (RF) (13).

**Figure 1:**
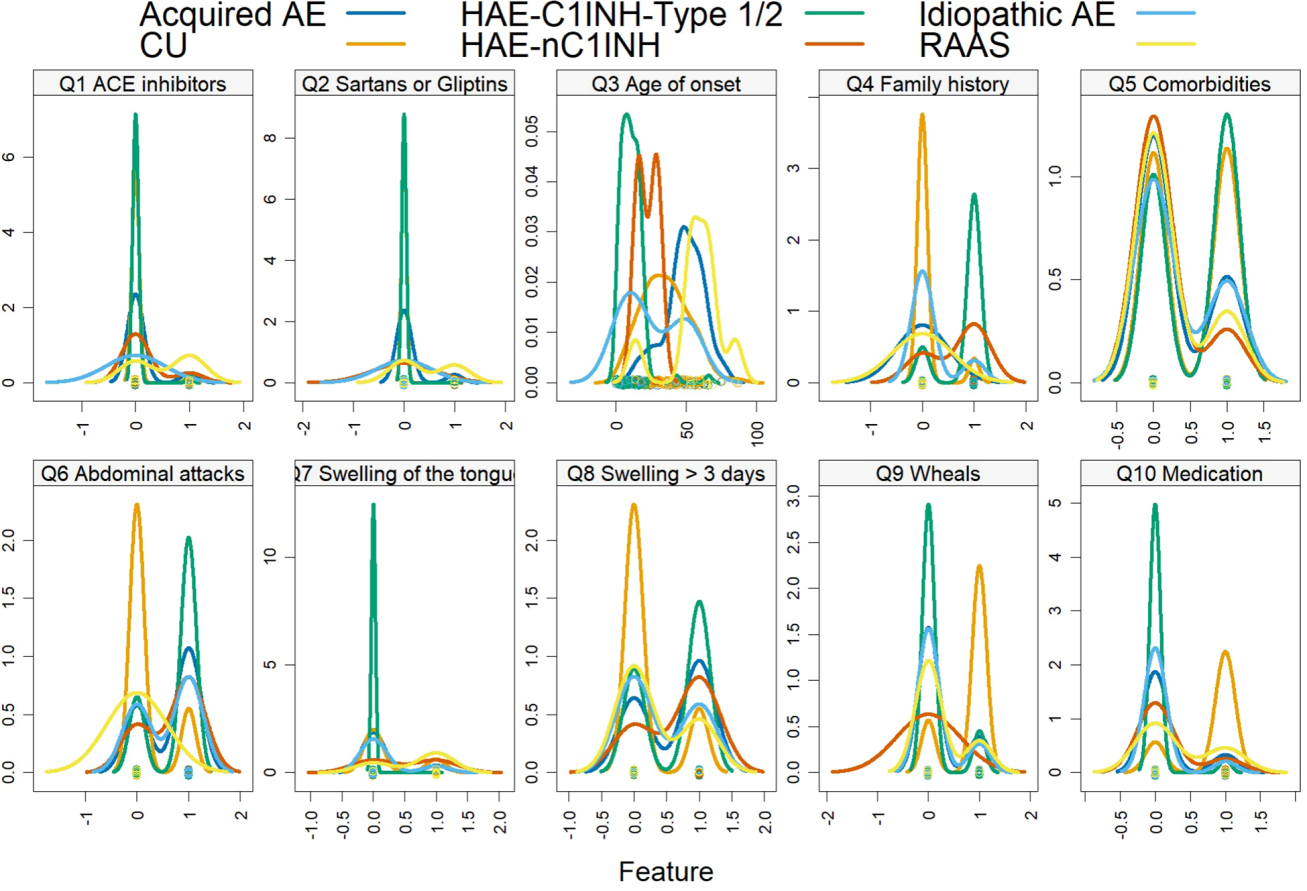
This image shows density plots for each of the ten questions (Q1-Q10). Most questions, except Q3, are binary (yes/no) questions. Each line represents a different disease condition, illustrating distinct distributions for each question. These unique patterns indicate that different conditions have specific response distributions for each question. By modeling these patterns, it is possible to diagnose based on the responses to the ten questions.

**Figure 2:**
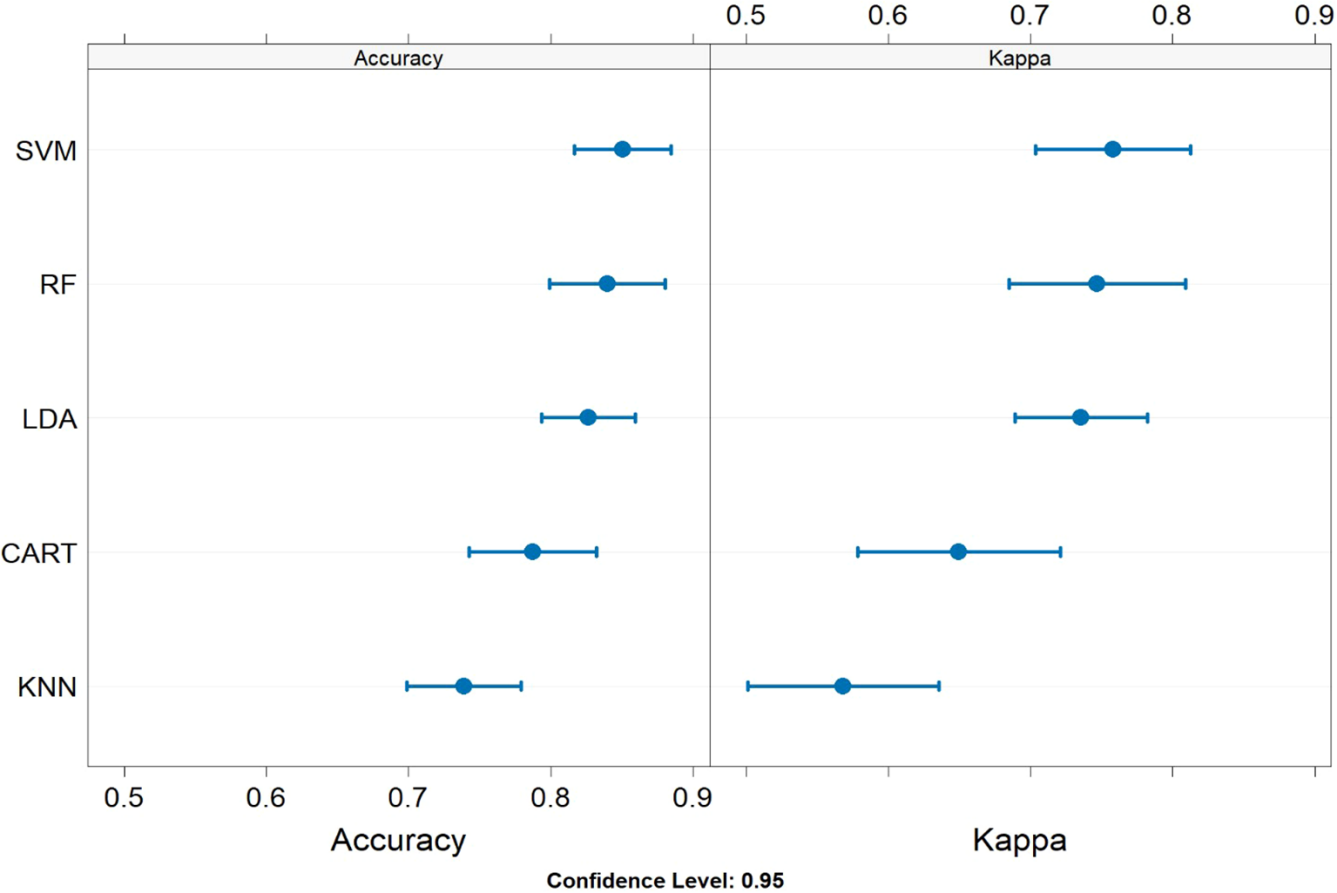
This plot shows the Percentage Accuracy and Kappa values for different models using their default settings. The models compared are SVM, RF, LDA, CART, and KNN. The SVM and RF models achieved the highest Percentage Accuracy and Kappa values, indicating superior performance.

The caret package of the R software environment was used to apply the defined algorithms. This platform allowed us to generate models with default settings (**Figure 1**) and to visualize data descriptively (**Figure 2**).

The random partitioning of the data was captured by a “seed” specified in R to enable replicability of the models. The surveys were randomly divided into test (20% of the data) and training (80%) data sets to generate those different models and ensure accurate validation. This randomization ensures that the model’s generalizability is appropriately tested across independent data subsets. The training set served as the basis for model generation. In contrast, the test data set was used to evaluate the predictive performance of each model and assess the reproducibility of each result. All data cleaning, processing and modeling steps were performed using the R statistical computing system (version 4.2.2; R Core Team, 2022).

#### 2.4.3 Metrics for performance evaluation

Percentage Accuracy and Kappa were used as the main estimates to evaluate the parameters of each model. These metrics were chosen because Percentage Accuracy provides a straightforward measure of correct classifications. At the same time, Kappa accounts for chance agreement, making it particularly suitable for evaluating the reliability of classification outcomes in medical diagnostics. The Classification rate (Accuracy) is the number of correctly predicted examples divided by the total number of observations (**Supplementary: Equation 1**). Cohen’s Kappa (Kappa) measures the agreement between two classifications, in this case, the actual outcome and the outcome predicted by the model (**Supplementary: Equation 2**).

#### 2.4.4 Hyper tuning of ML models Support Vector Machine and RF

We evaluated the model’s performance using key estimates: Percentage Accuracy and Cohen’s Kappa, each calculated on both the training and test datasets. Each metric ranges from 0 to 1, with a combined maximum total score of 4, representing optimal performance. This composite scoring approach allowed us to objectively compare models and select those that demonstrated the best balance between accuracy and reliability. Based on these values, we identified the SVM and RF models as the most promising candidates. While data cloning was explicitly avoided to prevent overfitting, we further optimized these models through rigorous hyperparameter tuning. This ensured that the models generalized well to unseen data, rather than memorizing the training set.

Based on their default settings, the two ML models with the highest Percentage Accuracy and Kappa values, SVMRadial and RF, were optimized through hyperparameter tuning. Various resampling methods from the caret package were tested and individually adjusted, such as boot, boot632, optimism_boot, cv, repeated-cv, Leave-One-Out Cross-Validation (LOOCV), and Leave-Group-Out Cross-Validation (LGOCV) (14). All resulting models were tested, validated, and subsequently optimized to improve prediction accuracy using functions like trainControl, tuneLength [ntree], and tunegrid [mtry].

Hyperparameter tuning for the RF model involved several steps. The resampling method was determined, and the “optimism_boot” method found the most stable values based on all runs using each parameter with different models. Next, “ntree” and “mtry” were evaluated (**Supplementary Figure 1**).

Hyperparameter tuning for SVMRadial was conducted similarly; the resampling method was optimized, with “repeated-cv” providing the most reliable results. The parameters “sigma” and “C” were evaluated (**Supplementary Figure 2**), demonstrating improvements in Percentage Accuracy and comparing different settings.

In the context of ML, a seed is a different training-test split, where a seed is a number used to initialize the random number generator, which ensures that the random partitioning of data and other random processes are reproducible. Using 900 different random seeds, we ensured the robustness of the optimization process and minimized the likelihood of selecting a model that performs merely by chance. The seed selection and transparent reporting of model parameters ensure that the study’s findings are fully replicable.

#### 2.4.5 Individual influence of the questions on the model

The Gini index is a method that uses the individual decision trees of the RF model to determine the influence of personal questions on the output. A higher Gini index means that the question generates more effective splits in the model, which increases the homogeneity of the resulting subgroups and thus has a stronger influence on the model’s prediction.

## 3 Results

### 3.1 Patient characteristics

**Figure 3:**
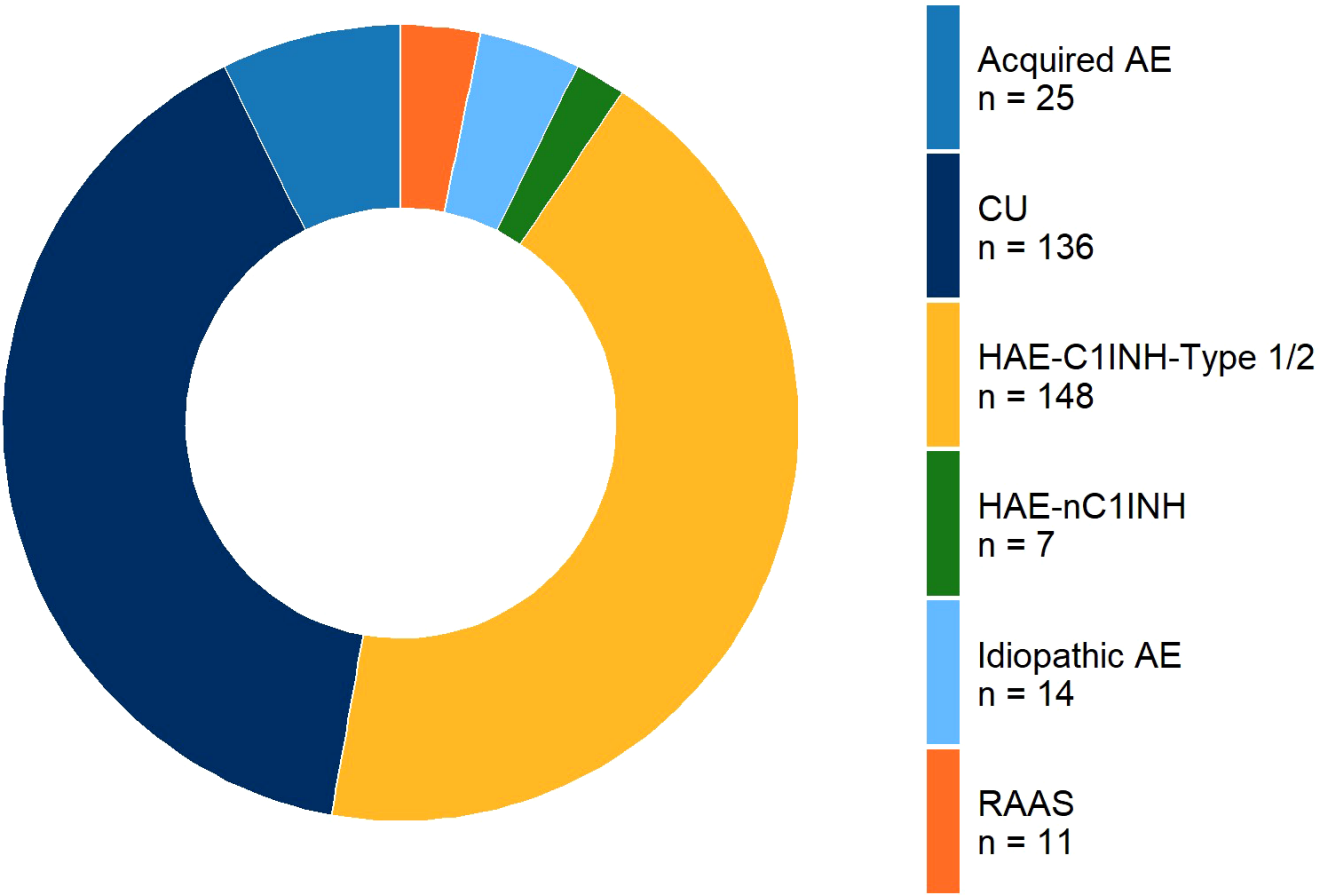
This pie chart displays the absolute values of the individual disease subtypes. The distribution of subtypes is as follows: HAE 1&2 (n=148), CU (n=137), Acquired AE (n=25), RAAS (n=11), Idiopathic AE (n=14), and HAE-nC1INH (n=7). The different colors represent each subtype, with the number of cases indicated for each category. Abbreviations: CU, chronic urticaria; HAE, hereditary angioedema; HAE 1&2, hereditary angioedema types 1 and 2; HAE-nC1INH, HAE with normal C1INH values; RAAS, renin-angiotensin-aldosterone system; AE, angioedema.

A total of 342 patients were included in the analysis; most patients surveyed (83%) had either CU (n=137) or HAE-C1INH-Type 1 or 2 (n=148; **Figure 3**). The remaining 17% (n=57) comprised idiopathic non-histaminergic angioedema (AE-UNK; n=14), drug-induced AE due to drugs targeting the renin–angiotensin–aldosterone system (AE-DI/RAAS: AE-ACEI, AE-Sartan and AE-Gliptin; n=11), acquired angioedema due to C1INH deficiency (AAE-C1INH; n=25), and HAE with normal C1INH (HAE-FXII and HAE-PLG; n=7).

The response patterns to the ten questions differed clearly between AE types (**Figure 1**). As expected, RAAS-associated AE showed “yes” answers almost exclusively in patients reporting current or recent use of ACE inhibitors and/or sartans or gliptins (Questions 1–2), whereas these medications were rarely reported in the other AE groups. A positive family history of recurrent angioedema (Question 4) was common in HAE-C1INH and HAE-nC1INH but uncommon in acquired AE, idiopathic AE, and RAAS-associated AE. Early onset symptoms (Question 3) were typical for HAE, intermediate for CU, and occurred later in life in acquired and drug-induced AE. Long-lasting swellings for ≥3 days (Question 8), severe abdominal attacks (Question 6), and swellings of the tongue (Question 7) were more frequent in the bradykinin-mediated HAE types, whereas a good response to antihistamines, corticosteroids or omalizumab (Question 10) and the presence of recurrent itchy wheals (Question 9) were mainly seen in CU.

### 3.2 The RF and SVM models were the best-performing ML algorithms for diagnosing AE types

Five different models were compared with the default model parameters of the caret packages; during this training process, the RF model had a Percentage Accuracy of 83·9% and a Kappa value of 84·9%. The SVM model had a Percentage Accuracy of 85·1% and a Kappa value of 84·9%. Both models demonstrated robust performance, with SVM slightly outperforming RF in Percentage Accuracy. These two models achieved the most stable results (**Figure 2**) and were trained with hyper-tuning. After hyperparameter tuning, the RF algorithm improved performance, making it the preferred model for further model training.

### 3.3 Hyper tuning results from the best settings of RF and SVM

The performance of the SVM and RF models was evaluated using training and test data from 900 different runs for each model.

For the SVM model, the mean Percentage Accuracy on the training data was 87·5%, with a mean Kappa value of 71.0%. On the test data, the mean Percentage Accuracy was 82·5%, and the mean Kappa was 79·8%, resulting in an overall sum score of 3·21. The SVM models showed a range in performance with a minimum overall sum of 3·0 and a maximum overall sum of 3·4.

In comparison, the RF model demonstrated higher performance across all metrics. The mean Percentage Accuracy for RF on the training data was 91·0%, with a mean Kappa of 85·9%. The test data showed a mean Percentage Accuracy of 86·1% and a mean Kappa of 77·5%, leading to an overall mean sum of 3·4. The range of the overall sum for the RF models was from 3·1 to 3·6.

These results indicate that the RF models consistently outperformed the SVM models, achieving superior accuracy and reliability across training and test datasets. The RF models also exhibited higher overall scores, indicating more reliable and effective performance than the SVM models.

### 3.4 Final RF model

The best result was achieved with the settings for mtry=4 and ntree of 500. With seed 708, the best model could be found, with a Percentage Accuracy of 90·8% (Kappa 85·3%) in the training data (**Table 2**) and 95·5% (Kappa 92·8%) in the test data set (**Table 3**). To evaluate the robustness of these results across different train–test splits, the performance of all 900 SVM and RF models is summarized in **Tables 4a**, **b**. These tables show that the RF models consistently achieved higher minimum, mean and maximum values for Percentage Accuracy and Kappa on both training and test data than the SVM models, resulting in higher overall sum scores for RF. This indicates that the superiority of the RF model is not limited to a single favorable split but reflects generally better and more stable performance compared with SVM.

**Table 2:** Confusion matrix training data: RF model seed 708; overall percentage accuracy = 90.76%, Kappa = 85.31%.

Training data confusion matrix	Acquired angioedema	CU	HAE C1INH-type 1/2	HAE-nC1NH	Idiopathic angioedema	RAAS	Class error
Acquired angioedema	7	7	1	0	3	2	0.65
CU	2	102	3	0	2	0	0.06422018
HAE C1INH-Type 1/2	1	5	112	0	1	0	0.05882353
HAE-nC1NH	0	1	5	0	0	0	1
Idiopathic angioedema	2	2	5	0	3	0	0.75
RAAS	2	4	1	0	0	2	0.77777778

Overall Percentage Accuracy=90.76%, Kappa=85.31%. CU, chronic urticaria; HAE, hereditary angioedema; RAAS, renin-angiotensin-aldosterone system.

**Table 3:** Confusion matrix test data: RF model seed 708; overall percentage accuracy = 95.45%, Kappa = 92.79%.

Test data confusion matrix	Acquired angioedema	CU	HAE C1INH-type 1/2	HAE-nC1NH	Idiopathic angioedema	RAAS
Acquired angioedema	5	0	0	0	0	0
CU	0	25	0	0	0	0
HAE C1INH-Type 1/2	0	1	29	0	1	0
HAE-nC1NH	0	0	0	1	0	0
Idiopathic angioedema	0	0	0	0	1	0
RAAS	0	1	0	0	0	2

Overall Percentage Accuracy=95.45%, Kappa=92.79%. HAE, hereditary angioedema; RAAS, renin-angiotensin-aldosterone system.

**Table 4a:** Evaluation of the SVM model based on training and test data from 900 different models.

SVM	Percentage accuracy (%) (training data)	Kappa (%) (training data)	Percentage accuracy (%) (test data)	Kappa (%) (test data)	Overall sum of each model
Min	86	58.35	74.24	77.45	3.04
Mean	87.47	70.98	82.46	79.84	3.21
Max	89.13	85.17	90.91	82.49	3.42

**Table 4b:** Evaluation of the RF model based on training and test data from 900 different models.

RF	Percentage accuracy (%) (training data)	Kappa (%) (training data)	Percentage accuracy (%) (test data)	Kappa (%) (test data)	Overall sum of each model
Min	88.65	82.09	75.76	60.37	3.14
Mean	91.04	85.87	86.14	77.47	3.41
Max	93.63	89.98	95.45	92.79	3.64

The “overall sum” of each model is calculated from the four values: *Percentage Accuracy (Training Data)*, *Kappa (Training Data)*, *Percentage Accuracy (Test Data)*, and *Kappa (Test Data)*. The overall score is the sum of individual parameters’ minimum, mean or maximum values.

The final modified ML RF model demonstrated a high degree of agreement between the disease types diagnosed by physicians and ML. An extremely high sensitivity (94% for both) and specificity (89% and 90%) were obtained for AE/CU and HAE type 1&2, respectively. Notably, the sensitivity of the ML-based diagnosis correlated with the sample size of each AE type, showing reduced sensitivity for rarer AE types. The sensitivity of ML-based diagnosis was linked to the number of patients per AE type, i.e., it was lower for types with fewer patients affected (**Table 5**).

**Table 5:** Statistical parameters training data: RF model seed 708.

Training data statistical parameters	Acquired angioedema	CU	HAE 1&2	HAE 3	Idiopathic angioedema	RAAS
Sensitivity	0.35	0.9358	0.9412	0.0	0.25	0.2222
Specificity	0.9725	0.8855	0.9038	1.0	0.9772	0.9925
Pos Pred Value	0.5	0.8430	0.8819	0	0.3333	0.5
Neg Pred Value	0.9502	0.9545	0.9527	0.9782	0.9662	0.9742
Prevalence	0.0727	0.3964	0.4327	0.0218	0.0436	0.0327

### 3.5 Individual influence of the questions on the model

Analysis of the Gini index across the individual questions revealed that Question 3 had the highest impact on the model’s predictive capability (Gini Index=57·51), whereas Question 5 had the least impact (Gini Index=3·26). The mean Gini Index decreases for each question in the final model shown in **Table 6**. Analysis of the mean decrease in Gini across the ten questions revealed that Question 3 (age at first occurrence of angioedema) had the greatest influence on the model’s predictions (Gini=57.51). This is expected from a modeling perspective, because Question 3 is the only continuous variable in the questionnaire and therefore offers many more possible split points than the other, mainly binary questions. The next most influential items were Question 10 (response to antihistamines, corticosteroids or omalizumab; Gini=34.63), Question 9 (recurrent itchy wheals; Gini=19.17) and Question 4 (family history of angioedema; Gini=18.94).

**Table 6:** Mean Gini decrease.

Mean Gini decrease	Acquired angioedema
Q1	5.39
Q2	3.49
Q3	57.51
Q4	18.94
Q5	3.26
Q6	7.95
Q7	5.26
Q8	5.54
Q9	19.17
Q10	34.63\

Clinically, this pattern is consistent with current knowledge: hereditary forms of AE typically start earlier in life than acquired or drug-induced forms (Question 3), mast-cell–mediated AE in CU is characterized by itchy wheals and a good response to antihistamines or omalizumab (Questions 9 and 10), and a positive family history (Question 4) points toward hereditary AE. Questions on specific drugs (Questions 1 and 2) and on the pattern of swellings (Questions 6–8) showed moderate Gini values and thus contributed to the classification but less strongly. Question 5, which inquired about prodromal symptoms, had the lowest importance (Gini=3.26), reflecting that these symptoms were relatively nonspecific in this dataset. The mean Gini values for all questions in the final model are summarized in **Table 6**.

## 4 Discussion

This study represents the first application of an ML model to support the differentiation of various AE types based on patient-reported responses. The model successfully identified patterns linked to six AE types and demonstrated promising alignment with expert assessments. However, it is important to emphasize that this tool is intended as a pre-assessment aid rather than a diagnostic system. It is designed to help guide patients in seeking appropriate medical consultation and to assist healthcare professionals in structuring their initial evaluations. It does not replace clinical expertise or formal diagnostic procedures.

Percentage accuracy was used as the primary evaluation metric to assess the model’s performance due to its simplicity and interpretability. However, given the class imbalance within the dataset, supplementary metrics such as Kappa were also considered to provide a more comprehensive assessment. Results from the confusion matrix confirmed that the model was well-fitted and did not exhibit signs of overfitting. Additionally, the feature importance analysis, measured via the mean decrease in the Gini Index, revealed that Question 3 (related to age) had the highest weighting, reinforcing its clinical relevance in AE differentiation. In contrast, Question 5, which pertained to a rare AE type, had the lowest weighting, underscoring the inherent challenges in predicting outcomes for conditions with limited data representation (**Table 6**). Given these findings, certain areas for potential improvement have been identified. While expanding the dataset with more cases of rare AE types would enhance the model’s accuracy, this remains challenging due to the limited data availability. Instead, future work could also focus on refining the model by exploring alternative analytical approaches to handle data imbalance more effectively. Further evaluations with different patient groups could also help assess the model’s applicability in various clinical contexts. However, rather than aiming for clinical implementation, the tool should continue to serve as a pre-assessment aid, guiding patients toward an appropriate medical consultation and supporting physicians in structuring their initial evaluations. To enhance accessibility, the model has been implemented on an online platform (https://tenquestions.net/), where it serves as a pre-assessment tool for individuals experiencing AE symptoms. This platform is intended to provide structured guidance based on patient-reported responses, helping users to seek appropriate medical expertise. However, it should not be used for self-diagnosis or as a substitute for professional medical consultation. Instead, it aids patients and physicians by offering an initial structured approach to AE-type differentiation.

Currently, the tool is available in multiple languages, with additional translations in progress to expand its accessibility further (English, German, Czech, Chinese, Turkish, Russian, Portuguese, French, Arabic, Italian, Spanish, Swedish, Lithuanian and Greek).

Despite its potential, the model faced several limitations, particularly regarding data imbalances. The disparity in patient representation across AE types—e.g., HAE-nC1INH (n=7) versus HAE-C1INH-type 1/2 (n=148)—impacted the accuracy of predictions, especially for rare AE types.

Traditional data-balancing techniques, such as over-sampling and under-sampling, were tested but did not improve performance, indicating the complexity of handling imbalanced datasets in ML models. This outcome suggests that traditional balancing techniques may not always be effective, particularly in complex and highly imbalanced datasets, emphasizing the need for alternative strategies or more sophisticated approaches to handle such challenges. Furthermore, exclusively AE-BK types of HAE-nC1INH, i.e. HAE-FXII and HAE-PLG, were included in the model and other mechanisms of HAE-nC1INH, i.e. AE due to vascular endothelial dysfunction (e.g. HAE caused by a mutation in the *MYOF* gene [HAE-MYOF] and HAE caused by mutations in the ANGPT1 gene [HAE-ANGPT]) were not considered.

Another key challenge was the potential risk of overdiagnosing certain AE types if they were overrepresented in the training data. While this study lacked sufficient data to mitigate such biases, future research should focus on integrating larger and more diverse datasets to improve the model’s robustness and generalizability.

The model’s accuracy is understandably lower for AE types with fewer patients. To address this limitation, collecting more data from patients with rare AE types in future studies will be essential, as this will likely increase the model’s diagnostic accuracy for these types. Additionally, the potential for diagnostic errors or patient misreporting, particularly in types with few patients, highlights the need for careful data validation and possibly integrating cross-verification methods in future studies.

The choice of an ML algorithm and hyper-tuning parameters may also be considered a limitation. Still, there is no guarantee that better results could be achieved with a different algorithm. However, this study’s comprehensive testing and optimization process suggests that the selected RF model is robust and well-suited for the current dataset. Future inclusion of more patients, particularly patients with rare AE types, will enhance the model’s robustness and generalizability.

Overall, the calculations in the ML model demonstrate high stability, making the RF model an optimal choice for our data. With the help of this model, a pre-assessment of AE type can be made with a high degree of accuracy. However, ongoing improvements and validation are essential to maintain and enhance the model’s performance as new data becomes available. Once optimized, the ten-question survey may be used in future diagnoses of patients with AE.

## Data Availability

The raw data supporting the conclusions of this article will be made available by the authors, without undue reservation.

## Supplementary material

Supplementary Table 1 AE nomenclature.
