# Supplementary material for: Classification of angioedema types using decision tree modeling
Source: Front Immunol. 2026 Jan 12;16:1697143. doi: 10.3389/fimmu.2025.1697143 (PMC12833243; doi:10.3389/fimmu.2025.1697143)
Supplement: Supplementary Table 1 — AE nomenclature. [file Supplementaryfile1.docx]

Supplementary Material

# Supplementary Figures and Tables

**Supplemental Figure 1:** Hyperparameter Tuning of Random Forest: Percentage Accuracy for Different ntree and mtry Values


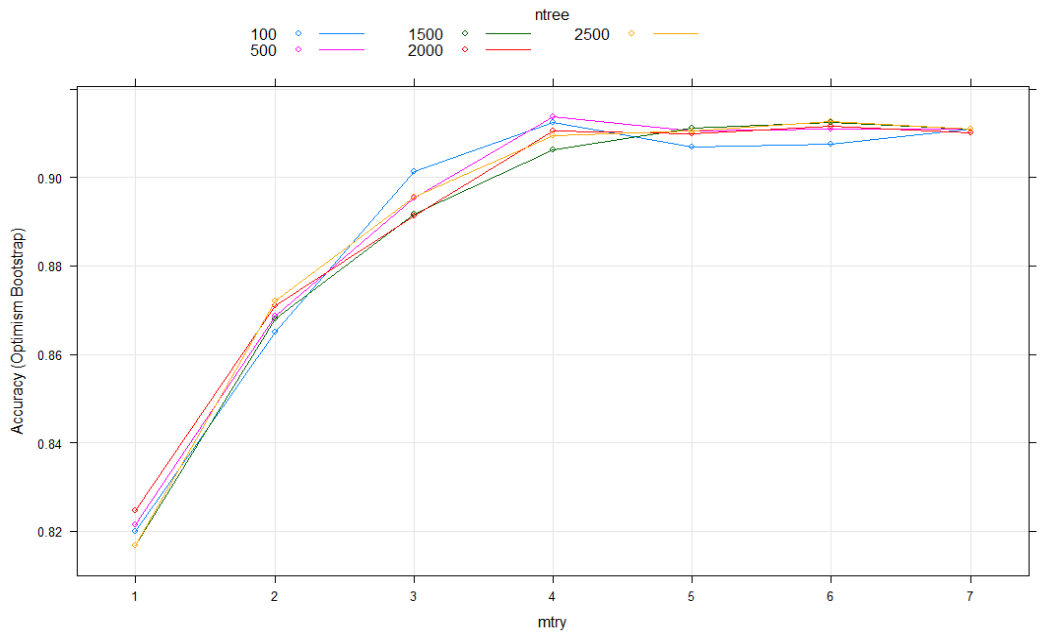


This plot illustrates the Percentage Accuracy values obtained using the "optimism bootstrap" method for different combinations of the "ntree" and "mtry" parameters in the Random Forest (RF) model. Each line represents a different "ntree" value, while the x-axis shows the "mtry" values. The results, each derived from 30 iterations, indicate that Percentage Accuracy generally increases with higher "mtry" values, peaking around 4. Notably, the model with 500 trees (ntree) achieves the best Percentage Accuracy across different "mtry" values.

**Supplemental Figure 2:** Hyperparameter Tuning of SVM: Mean Percentage Accuracy for Different Sigma and C Values


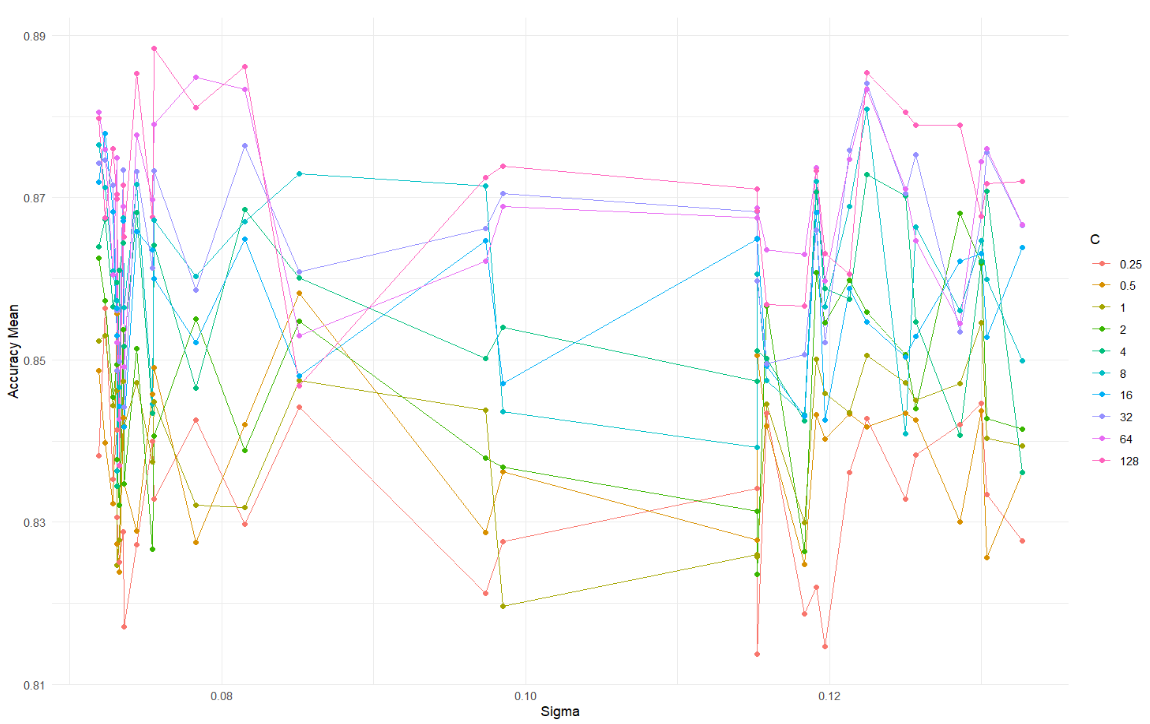


This plot illustrates the mean Percentage Accuracy values for different combinations of Sigma and C parameters in the SVM model. Each mean Percentage Accuracy value is derived from 30 iterations, ensuring robust results. The lines represent varying C values, while the x-axis shows Sigma values. The results indicate significant variability in Percentage Accuracy depending on these parameter combinations. Sigma values around 0.08 to 0.12 generally achieve higher Percentage Accuracy, with noticeable peaks and troughs indicating sensitivity to changes. Higher C values (such as 128) tend to yield better Percentage Accuracy, but stability varies significantly.

$$Eq1: Percentage Accuracy=\frac{\mathrm{TP}+\mathrm{TN}}{TP+TN+\mathrm{FP}+\mathrm{FN}}$$

TP: True Positives (correctly predicted positives)

TN: True Negatives (correctly predicted negatives)

FP: False Positives (incorrectly predicted positives)

FN: False Negatives (incorrectly predicted negatives)

$$Eq2: Kappa=\frac{p_{o}-p_{e}}{1-p_{e}}$$

$p_{o}$​: The observed agreement, calculated as the number of instances correctly classified by both classifiers divided by the total number of instances.

$p_{e}$​: The expected agreement, calculated based on the marginal totals of the confusion matrix.^1^

**References**

1. McHugh ML. Interrater reliability: the kappa statistic. Biochem Med (Zagreb) 2012;22(3):276-82. (In eng).

**
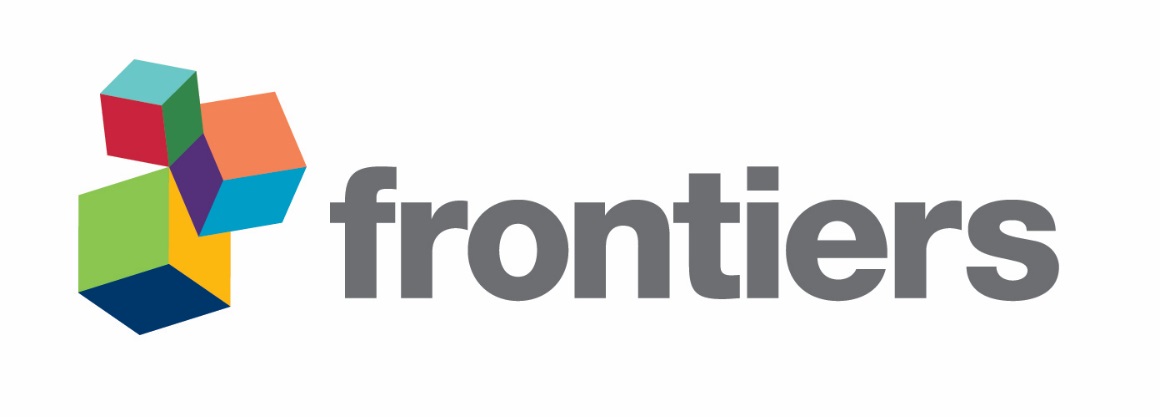
**
