# Supplementary material for: Classification of angioedema types using decision tree modeling
Source: Front Immunol. 2026 Jan 12;16:1697143. doi: 10.3389/fimmu.2025.1697143 (PMC12833243; doi:10.3389/fimmu.2025.1697143)
Supplement: Supplementary file 2 [file Supplementaryfile2.docx]

**Supplement Table 1:** AE nomenclature

| **Nomenclature old, acronym** | **Nomenclature new, acronym** |
| --- | --- |
| Acquired angioedema, AAE | Acquired angioedema due to C1INH deficiency, AAE-C1INH |
| Chronic urticaria, CU | Angioedema due to urticaria, AE-URT |
| Hereditary Angioedema due to C1 inhibitor deficiency, HAE C1INH-Type 1/2 | HAE due to C1INH deficiency, HAE-C1INH |
| Hereditary angioedema due to normal C1INH, HAE-nC1NH | Hereditary angioedema due to normal C1INH, HAE-nC1INH. At the time of drafting the study presented here only HAE-FXII and HAE-PLG were known |
| Idiopathic angioedema | Angioedema due to unknown cause, AE-UNK |
| Angioedema due to intake of drugs targeting the renin-angiotensin-aldosterone system, RAAS | Drug-induced angioedema (AE-DI) due to intake of drugs targeting the renin-angiotensin-aldosterone system (RAAS, i.e. AE-ACEI, AE-Sartan and AE-Gliptin, which are presumably BK-driven) |
